# The Causes and Treatment of Chronic Gonorrhea

**Published:** 1893-06

**Authors:** J. Henry Dowd

**Affiliations:** Late House Surgeon Sisters of Charity Hospital, also of United States Marine Department; Fellow of the Buffalo Academy of Medicine; Cor. Broadway and Michigan Streets


### THE CAUSES AND TREATMENT OF CHRONIC GONORRHEA.

By J. HENRY DOWD, M. D.,

Late House Surgeon Sisters of Charity Hospital, also of United States Marine Department; Fellow of the Buffalo Academy of Medicine.

*Mr. President, Ladies and Gentlemen:*

In choosing a subject for my paper, I have endeavored to select one which we meet almost daily in our practice; especially can this be said of the younger members of the profession, to whom most of these cases apply for treatment. I know of no disease that will cause you more anxiety, and in yourself furnish a subject of neurasthenia quicker, than to have a patient present himself day after day with the same tune, "I am about the same." This, of course, causes you to seek your authority for further advice, which you use most faithfully, only after the lapse of a week or a month to hear the familiar sentence, "I am about the same." You become discouraged, the patient also, and before you are aware he slips into the hands of another.

In regard to specialists in this branch of surgery, or, more strictly, venereal diseases in general, I cannot advise you to become such, although a professor of anatomy in a large London school used to, and probably does yet, say to his class when lecturing on the reproductive organs: "Gentlemen, there is more money made from this region of the human body than from all the rest together." Men have worshiped at the altar of Venus since the world began, and they will undoubtedly worship there until it ends. Venereal diseases are described in the Bible and they will probably exist until the day of judgment. I cannot give statistics, but I am safe in saying that one-third of the gynecological diseases today can be dated from the first connection with one who has erred in his youth, and to this important subject medical colleges usually give one or two lectures a year. The question is often asked, How soon does gonorrhea become chronic? Any time after five or six weeks. Personally, I believe clap can be cured in less time than that, but after that it may be considered chronic.

The causes of this disease are almost as numerous as the remedies, and I believe it is folly to apply any remedy until the cause be known, for what benefit can you expect from an injection, let it be ever so strong, or the latest on the market, if a contracted meatus or stricture be present? The first and most important cause of long continued discharge is where the surface is denuded of epithelium and covered with granulations; and this you can see is the first step to stricture. These granulations, projecting as they do into the canal, diminish its caliber, the urine striking this spot is thrown backwards in confusion, causing irritation of the mucous membrane, and from here arises the discharge. Now, allow this to continue for month after month, the deeper structures become involved, the granulations harder, a cicatrix is formed, and stricture is the result. The diagnosis of granular urethra is not always easy, but by a good history, examination of the urine use of the bougie ã boule, and endoscope, it is usually quite possible to locate the trouble at one or more spots. The patient tells you his urine contains threads, or he can feel a drop start, say two, three, or four inches from the meatus and come forward. Now, having him urinate in two separate clean glasses, if the first contains débris, and the second is clear, you may be certain it is the anterior urethra you have to deal with. Our next aid is the bougie, selecting the largest one that will pass the meatus (providing it is not contracted); gently pass it down the canal, noting any spot or spots which may give the patient pain, and measure the distance for further use. The endoscope I will give an opinion of, by quoting from a well-known author: "If one can afford this pretty plaything, the effect is certainly very taking. The glistening tubes, with reflector, lamp and long lines of bright, covered wires, as well as the sensational idea of a light being shed inside the body, has a great moral effect and will, no doubt, do the practitioner no harm if it does not do the patient any good." I will change the last sentence to read: If it does not aid in diagnosis, for I consider the endoscope indispensable in applying treatment.

The trouble being now located, the distance which we previously found must be measured on the endoscopic tube, the latter being passed in until the end rests against the diseased spot. The obturation now being withdrawn, the application can be made. Silver nitrate is probably the best remedy, and can be used in a 1 to 20 per cent. solution, as there is more or less inflammation behind these granulations, and the application causing an acute one, I order an injection consisting of muriate of hydrastine, grains 10 to 15, to 4 ounces of water, bismuth and glycerine. Usually, one application will suffice, but certain cases may call for three or four, to be made at intervals of two or three days, always increasing the strength of the solution. I will not mention other solutions, for if there is to be any result it will be obtained with silver.

The second most common cause is a contraction, in any part of or the whole length of the canal, thus diminishing its caliber and giving rise to a pathological condition similar to the one already mentioned. The diagnosis of this condition is comparatively easy, for with the eye we see the contracted meatus, with the bougie or Otis urethrometer detect the stricture, and if the discharge is very thick, whitish yellow, and abundant, chronic inflammation of the whole tract is probably present. The meatus, if small, must be cut to an extent sufficient to admit a No. 16 Eng. sound, which should be passed into the urethra. This not only acts as a tonic, but serves to separate the cut surfaces. The patient must be instructed to pass a hair-pin (in the way shown) into the urethra in at least twelve hours, to be repeated in fifteen to twenty hours thereafter. Unless this is done, the cut surfaces will be tightly closed in a day or so. If by the use of the bougie stricture is found, this must be cut or dilated. When chronic inflammation exists, the only treatment is the passage of a full-sized sound, leaving it in place for five minutes or more. With the above conditions I always give an injection similar to the one already mentioned.

The third cause is the rough use of instruments and the squeezing to which some patients submit the organ every time they urinate. This should hardly be called a cause, but I consider it sometimes very important, having seen patients who upon being advised to let the doctor do the squeezing, and along with proper remedies, recovered rapidly. Who of you, treating a person for conjunctivitis, would allow him to squeeze and rub his eyes four or five times daily, to see if there was any discharge? I may say no one. Then, have the urethral mucous membrane treated as gently, for it is just as delicate as the conjunctiva.

The fourth condition is inflammation of the lacuna magina, or the numerous pockets along the canal. Our only aid in diagnosis is the passage of a sound, which presses the discharge from these pockets, and on withdrawal of the instrument the discharge follows. The best and only treatment is the passage of a full-sized sound every alternate day and the use of an astringent injection. A few cases are recorded where the lacuna magina, being found the seat of trouble, has been slit open, thus making an open instead of a closed sac.

The fifth cause is an inflammation of Cowper's, or of the prostate gland with post urethritis. The glands of Cowper when affected will manifest themselves by an enlargement on either side of the perineum posterior to the scrotum. Incision externally, and packing with gauze will eradicate this condition. Prostatitis with post urethritis is not always easy of diagnosis, but certain symptoms point directly to that locality, namely, if a sound being passed causes great pain near the bladder, or if during ejaculation of semen there is pain. If in the course of the disease there has been epididimitis or cystitis, and by pressure on the gland it is tender, the cause may be pretty positively located here. The treatment of this condition is somewhat discouraging, and you can only hope for a good result by long continuation of the same. Counter-irritation to the perineum sometimes acts well. A sound should be passed, and left in for a few minutes, and on its withdrawal the retrojection catheter passed to the neck of the bladder, and from one to two quarts of hot water, impregnated with some astringent, allowed to pass over the entire mucous tract. After this, with a Keys or Ultzmann deep urethral syringe, an injection can be placed in this region. I generally use aristol nitrate, in a 1 to 10 per cent. solution, allowing one or two drops to enter the canal, increasing at each application. This treatment may have to be repeated, at intervals of two or three days, for some time, in the meantime having the patient use an injection, which must be worked well back into the posterior urethra. In this condition, occasionally, good results are obtained by giving tincture cantharides, in two or three-drop doses, three times daily.

Now, let me speak of the prime factor in most all cases; and sometimes the only one. I refer to debility. We all know that twenty-four out of twenty-five cases of gonorrhea apply to a druggist for treatment, or use the favorite prescription of a friend, and what do they use ? Simply copaiba and cubebs, which is continued sometimes for months, or until the stomach absolutely refuses to tolerate another drop, or the characteristic copaiba rash appears, thus forcing them to see a doctor, thinking, possibly, they may have syphilis. You find the man with his appetite paralyzed, so to speak; what food he does take passes through the alimentary canal, untouched by the digestive fluids; bowels in an obstinate, constipated condition; the nervous system in a state of excitement; in short, a good subject of nervous prostration. This condition must be ascertained by the medical man, and treated accordingly by tonics of iron, strychnine, pepsin, good hygienic surroundings, etc. I can call to mind one case of four years' standing, which was cured in three days by iron, pepsin, strychnine, and cascara sagrada; another, of over a year's duration, by cod-liver oil; and a third, by treating the man for his syphilis.

Before closing, I want to speak of one condition which I have met twice in the past three years, and which may be called hyperesthesia of the urethral mucous membrane, and due to ungratified desire. The first case was in a married man who contracted gonorrhea and kept it twenty months. I found a small ulcer one-half inch from the meatus. This I treated by suppositories of aristol and bismuth, the pus discharge stopping in four or five days, but a thin, watery secretion escaping, and a constant itching and tickling sensation along the canal, which continued for some time, or until I advised him to renew his marriage relations, which he did, reporting in two or three days that he was well, and now, in over a year, he has had no return. I suppose he has been doing his duty ever since. I compared him to a person who being fond of a certain article of food which he has not partaken of for some time, suddenly, in passing a hotel or restaurant, the odor from the much fancied article reaches his Schneiderian membrane, and the result is the "mouth waters." I do not wish to infer that the urethral mucous membrane is furnished with the sense of smell (although, as a prominent surgeon remarked, it would be a good thing if it were), but, through the brain, it is kept in an over-sensitive condition unless relieved.

The second case was an almost similar one to the first, but, remember, these cases are few, and where you may be successful in giving such a prescription to one person, to another you may light up his symptoms acutely, or cause some poor female to become an invalid for life by ovaritis, salpingitis, or peritonitis, and even death. It was a true saying which, I think, was uttered by Dr. Joseph Price, of Philadelphia: "Clap kills more people than syphilis, although syphilis is a disease of a lifetime."

COR. BROADWAY AND MICHIGAN STREETS.

